# Understanding the Workflows in Non‐Guided and Static Computer‐Assisted Implant Surgery

**DOI:** 10.1002/cre2.70309

**Published:** 2026-02-19

**Authors:** Adria Jorba‐Garcia, Emilio Couso‐Queiruga, Clemens Raabe, Krzysztof Chmielewski, Michael Bornstein, Bilal Al‐Nawas, Nikos Mattheos

**Affiliations:** ^1^ Department of Oral Surgery and Implantology, Faculty of Medicine and Health Sciences University of Barcelona Barcelona Spain; ^2^ Department of Oral Surgery and Stomatology, School of Dental Medicine University of Bern Bern Switzerland; ^3^ Department of Oral Surgery and Implantology Goethe University, Carolinum Frankfurt am Main Germany; ^4^ Private Practice Gdansk Poland; ^5^ Department of Oral Health & Medicine, University Center for Dental Medicine Basel UZB University of Basel Basel Switzerland; ^6^ Department of Oral and Maxillofacial Surgery University Medical Center Mainz Mainz Germany; ^7^ Implants and Esthetic Restorative Dentistry Centre, Faculty of Dentistry Chulalongkorn University Bangkok Thailand; ^8^ Department of Dental Medicine Karolinska Institute Stockholm Sweden; ^9^ Faculty of Dentistry The University of Hong Kong Hong Kong

**Keywords:** computer‐assisted implant surgery (CAIS), dental implants, digital dentistry

## Abstract

Contemporary implant dentistry aims to achieve long‐term biological, esthetic, and functional successful outcomes. Thus, instrumental to this aim is designing an integrated system in which the supported prosthesis & components, anatomical and phenotypical tissue characteristics, and the fixture act synergistically to maintain peri‐implant tissue stability and health. Central to these goals is (1) the definition of a patient‐optimized three‐dimensional (3D) implant position during comprehensive treatment planning and (2) the intraoperative transfer of the plan to the final implant position. Preoperative planning is supported by digital imaging, primarily via tomographic and surface scans of the patient's anatomy, and subsequent data processing in dedicated planning software, allowing for comprehensive case evaluation. The digital treatment plan which initiates computer‐assisted implant surgery (CAIS), can be executed by means of non‐guided or guided implant surgery approach; the latter involving static, dynamic, and robotic techniques. This white paper aims to provide a comprehensive overview of the required resources and workflows involved in digital implant treatment planning and subsequent implant placement using non‐guided and static CAIS approaches.

## 1 Introduction

As the focus of implant dentistry has shifted toward achieving long‐term biological, esthetic, and functional outcomes, an increasing number of studies have highlighted the critical role of digital treatment planning and guided implant surgery in minimizing intra‐ and postoperative complications (Katafuchi et al. 2018), enabling prosthetically driven implant positioning, with the ultimate goal of maintaining peri‐implant health and adequate treatment outcomes (Janda and Mattheos 2024). Consequently, it has become evident that successful implant therapy relies on the local ability to design an integrated system in which a well‐designed implant‐supported prosthesis, an optimally positioned implant fixture, adequate peri‐implant mucosa dimensions, and other anatomical characteristics promote peri‐implant tissue stability in an environment with continuous presence of oral biofilm (Janda and Mattheos 2024; Pedrinaci et al. 2024a; Avila‐Ortiz et al. 2020; Tavelli et al. 2021).

Computer‐assisted implant surgery (CAIS) can be essentially defined as the use of computer technologies, dedicated software, and specialized devices to assist in transferring a preoperatively defined case and patient‐optimized three‐dimensional (3D) implant position to the surgical field (Jorba‐Garcia et al. 2025), following a digitally created treatment plan.

In non‐guided (ng‐CAIS) scenarios, the digital treatment plan is used by the surgeon to intraoperatively identify the 3D implant position in the surgical site using local measurements, anatomic landmarks, or analog aids such as intraoperative prosthetic templates. On the other hand, guided CAIS utilizes digital aids and technology to assist accurate implant placement and can be further divided into static (s‐CAIS), dynamic (d‐CAIS), and robotic (r‐CAIS). Although d‐ and r‐CAIS have documented significant advances recently, s‐CAIS remains by far the dominant modality for guided implant placement (Yeo et al. 2025a) while the majority of implants at present are still being placed with non‐guided protocols (Raabe et al. 2025).

The aim of this white paper is to provide a comprehensive overview of the fundamental principles and concepts of CAIS across the different approaches, as well as the essential shared steps of the workflow. As part of the ITI White Papers the paper is intended to provide structured orientation rather than exhaustive technical instruction and hopefully introduce clinicians to concept that would be further analyzed in more specific works. The readers are advised to navigate the presented workflows with the support of the Gloassary of CAIS and related terms (Jorba‐Garcia et al. 2025), which is an essential complement of the content in this paper. This paper will focus on describing detailed workflows for ng‐ and s‐CAIS, in particular concerning the steps and technologies involved for different clinical scenarios, from single tooth gaps to full edentulism. Finally, this paper will aim to offer a comprehensive overview of the potential and limitations, cost‐related factors, as well as indications/contraindications and other parameters relevant to clinical decision making.

### 1.1 Diagnostics Essentials and Data Collection

In the past, implant position planning was often conducted on 2D intraoral or panoramic radiographs and mainly driven by local anatomy, with prosthetic considerations relegated to a secondary role. However, the widespread introduction of cone‐beam computed tomography (CBCT) and dedicated planning software has enabled comprehensive case assessment and appropriate preoperative 3D implant positioning (Bornstein et al. 2015).

CBCT can provide high‐definition images with low voxel sizes, allowing a sub‐millimeter resolution ranging from 0.4 mm to as low as 0.075 mm. Generally, a voxel size of 200 µm is sufficient for implant planning. However, smaller voxel sizes may be considered when finer anatomical details need to be visualized or segmented (Jacobs et al. 2018a), although this potentially increases radiation dose. The level of radiation dose of CBCT in implant dentistry should follow the ALADAIP principle (As Low As Diagnostically Acceptable, being Indication‐oriented and Patient‐specific).

Additionally, the datasets obtained from CBCT can be processed and segmented in a non‐orthogonal manner, allowing for the creation of oblique or curved planar reconstructions, for example, in cases of simulated panoramic images (Jacobs et al. 2018b; Fuglsig et al. 2024). Furthermore, this method enables the production of serial cross‐sectional planes, offering detailed views of the region of interest (ROI) from multiple angles. Such capabilities enhance diagnostic precision, enabling clinicians to better evaluate the prospective surgical site.

Evaluation of the periodontal phenotype and alveolar ridge morphology becomes crucial. While CBCT provides valuable information of alveolar bone morphology on edentulous site, as well as bone morphotype and tooth anatomy in different planes, reliable CBCT‐based 3D‐surface reconstruction based on Hounsfield units is not feasible. Tooth anatomy and thin phenotypes, in particular, may be inadequately represented, especially regarding the buccal bone plate (Schulze et al. 2024). Thus, an optical surface scan could supplement detailed information on the dental anatomy and the soft tissue phenotype, yet it will not improve the measurements on hard tissue phenotype (Couso‐Queiruga et al. 2021, 2023). This scan can be obtained through an intraoral scan (IOS) or by digitizing analog dental casts using a desktop optical scanner.

The digital implant treatment planning requires a set of DICOM (Digital Imaging and Communication in Medicine) files from a CBCT, ideally supplemented by an STL (Standard Tessellation Language), OBJ (Wavefront OBJ), or PLY (Polygon File Format) file from a surface scan of the patient's dental arches and occlusion. Superimposing DICOM and STL/OBJ/PLY files with accuracy, a process known as 3D data registration (Jorba‐Garcia et al. 2025), provides the clinician with detailed information about the anatomic conditions of both soft and hard tissue and an improved understanding of the occlusion by aligning the files within the same coordinate system (Pedrinaci et al. 2025).

The precision of data registration is crucial for the success of CAIS procedures, yet it is often overlooked. Accurate superimposition requires CBCT scans to be acquired under specific conditions that may differ from standard imaging protocols. This process can be compromised by various imaging artifacts, including metal artifacts from dental restorations or motion‐related artifacts. In partially or fully edentulous patients, the availability of reliable registration points becomes particularly important and may create the need for radiographic templates. Therefore, before acquiring the CBCT, clinicians must select a field of view (FOV) that encompasses the ROI and ensures the predictable identification of a sufficient number of registration points. This can be facilitated by blocking the bite (teeth out of occlusion), keeping the tongue away from the palate, and using lip retraction during imaging (Januário et al. 2008). Cotton rolls are commonly used for this purpose (Lanis et al. 2017); they could however, introduce radiopaque artifacts. Lip and cheek retractors such as Optragate (Ivoclar Vivadent AG, Schaan, Liechtenstein) can be used.

#### 1.1.1 Optional/Additional Steps

Although not always essential, preoperative data collection could include 2D photography, 3D facial scanning, and dynamic digital occlusion registration and articulation (Amin et al. 2023; Revilla‐León et al. 2023). These methods could be especially useful when planning a full‐arch rehabilitation or a bimaxillary rehabilitation, since valuable information related to esthetics and functionality can be obtained from these tools. All these 3D data sets can be registered and aligned with the other essential, previously acquired data (i.e., surface scan and CBCT images), creating a “virtual patient” with multiple layers of imaging.

### 1.2 Digital Implant Treatment Plan ‐ Computer Assisted Design (CAD)

The modern, design‐driven paradigm of implant therapy starts with the design of the implant‐supported prosthesis. Planning then continues with the optimal configuration of the implant supracrestal complex, and only as the final step does it identify the optimal position and dimensions of the implant, focusing on the design of an entire system in interaction with the peri‐implant tissue and bacteria. Several CAD implant planning software (CAD‐IPS) systems have been developed to facilitate the diagnostic and preoperative planning phases of implant surgery, covering a wide array of functions.

Initially, the CBCT data set is imported into the software and adjusted to the specific needs, including 3D CBCT data segmentation, which may help in the identification of the respective anatomic structures by isolating and creating 3D objects of teeth, bone, or structures such as the maxillary sinus. Segmentation can be conducted manually, automatically using predetermined density values, or increasingly with artificial intelligence (AI) tools included in some CAD‐IPS. Subsequently, the superimposition of the STL/OBJ/PLY files (3D data registration) is conducted, allowing the correct orientation and spatial relationship of all depicted anatomic structures. Thereafter, a virtual prosthesis might be visualized through different pathways: (a) digital libraries of teeth/components, (b) AI‐based generation, and (c) smile‐design tools or externally designed digital set‐ups.

CAD‐IPS platforms may provide additional functions such as the delineation of the mandibular canal, virtual tooth extraction, warning in cases of anatomical structures proximity, linear and angular measurements, or inter‐implant angle measurement and parallelization.

#### 1.2.1 Essential Workflow for Partial Edentulism

**Figure 1:**
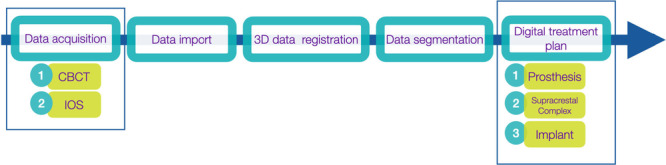
Workflow for digital implant treatment plan in partial edentulism.

**Figure 2:**
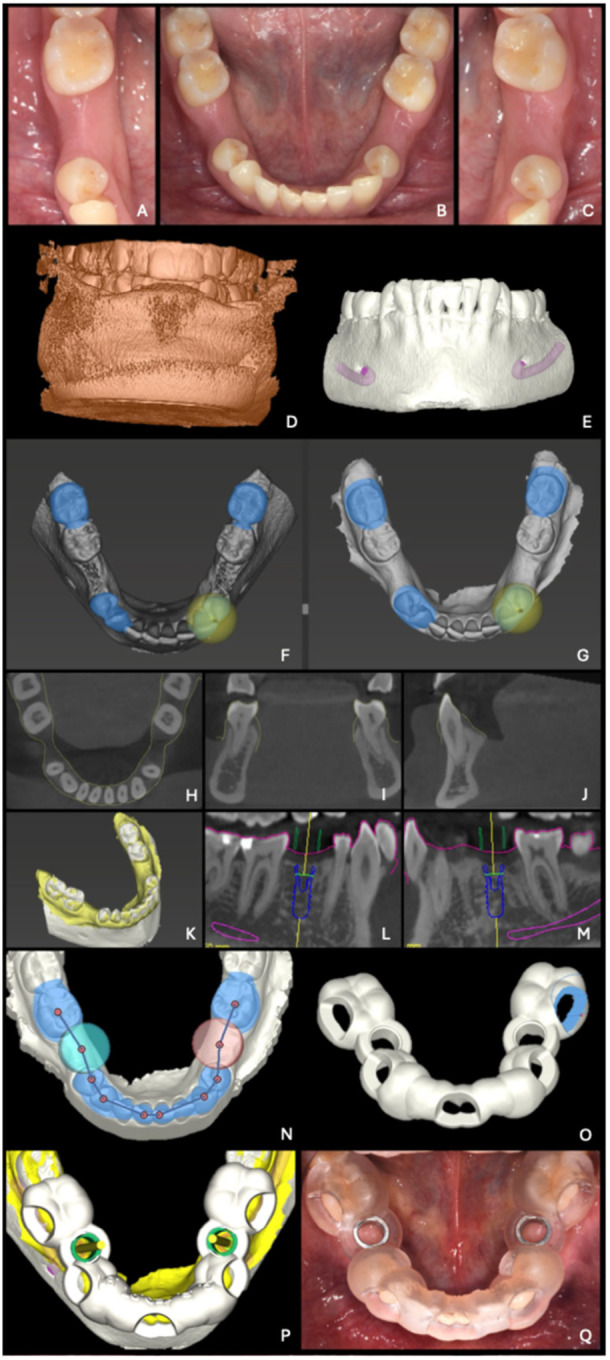
Clinical and radiographic workflow enabling digital implant planning in partially edentulous patients. A, B, C: Occlusal views of the mandible and bilateral single edentulous second premolar sites. D: Blocked‐bite cone‐beam computed tomography under soft‐tissue retraction. E: Segmentation of the mandible and the mandibular canal. F, G: Data registration and critical evaluation in axial (H), coronal (I), sagittal (J), and 3D (K) views. L, M: Virtual implant positioning. N: Definition of guide extension. O: Design of buccal fenestrations. P, Q: Virtual design of the guide and corresponding clinical presentation after surgical guide manufacturing.

Workflow for digital implant treatment plan for partial edentulism is summarized in Figure 1 and depicted in a clinical case in Figure 2.

1. Data acquisition:
  a. CBCT scan of the patient. Ideally, the scan should focus on the ROI and optionally include registration devices, such as radiopaque set‐ups, in extended edentulous spans. However, for accurate data registration, it may be necessary to extend the field of view to include several teeth or anatomical landmarks. For reliable 3D data registration, a full‐jaw scan is recommended (e.g., 8 × 8 cm, 10 × 10 cm, 5 × 10 cm, or 5 × 8 cm). Special attention should be given to minimizing patient movement to prevent motion artifacts (Hamilton et al. 2022).
  b. Surface scan of dental and oral tissue (IOS or extraoral, i.e., digitalization of dental casts, or radiopaque set‐ups).
2. Data import: Data are imported into the CAD‐IPS software for implant planning.
3. Data Segmentation: If desired but not mandatory, the DICOM dataset could be transformed into an object (i.e., STL file). This 3D data segmentation could be performed on the entire anatomy captured by the CBCT or only on a desired structure (i.e., teeth, the sinus, or the maxillary or mandibular bone).
4. 3D data Registration: Accurate alignment of the DICOM and STL files within one coordinate system. This process is typically facilitated by the CAD software by identifying manually at least three identical landmarks/fiducial points in both data sets (Pedrinaci et al. 2025; Flügge et al. 2017). In the past, these points were selected manually in the 3D reconstructed CBCT and STL files, but most modern CAD‐IPS allow for an AI‐assisted alignment without the need for manual selection of landmarks in both images (Ntovas et al. 2024; Elgarba et al. 2024a). Finally, the antagonist scan may also be imported and aligned according to the patient's occlusion in a static position, typically at maximum intercuspidation.
5. Digital treatment plan: The entire implant system (prostheses, implant supracrestal complex, and dental implant) is planned according to the patients' needs, local anatomic and phenotypical characteristics, and the best available evidence. This process is “top‐down,” starting with the design of:
  a. The digital prosthesis, also called digital set‐up, is guided by esthetics, function, occlusion, the local anatomic and phenotypic characteristics, and the patients' needs/expectations.
  b. The Implant Supracrestal Complex or the transmucosal part of the implant‐prosthesis system, including the design of the emergence profile, its dimensions (i.e., height and width), and its relationship with the peri‐implant phenotype.
  c. The implant position and dimensions required for the optimal rehabilitation supporting the designed supracrestal complex and prosthesis, including implant type, design, dimensions, and surface.

The implant position described above would constitute the “prosthetically‐driven implant position”.

d. Cumulation of the above into a patient‐optimized position (also known as Bio‐Restorative Implant Position (Pedrinaci et al. 2024b); Erratum in: J Esthet Restor Dent 2025): Although it is widely accepted that the optimal prosthetic position is a primary aim of the digital treatment plan, the final implant position must also ensure osseointegration, surgical feasibility, and minimal invasiveness, respecting local and phenotypical characteristics, biology, and safety. When these parameters are appropriately combined with the optimal prosthetic position through digital planning and clinical judgment, the result is a personalized, precise position, tailored to the patient's unique needs.

#### 1.2.2 Workflow for Immediate Implant Placement

If implant placement is intended in a fresh post‐extraction socket, an additional step in the workflow of partial edentulism would be the “virtual tooth extraction”, which involves the removal of the to‐be‐extracted tooth from the digital treatment plan after segmentation. This step allows better visualization of the hard tissue morphology.

#### 1.2.3 Workflow for Full Edentulism

Certain modifications of the workflow should be considered during data acquisition and treatment planning for edentulous patients. From a workflow point of view, we can differentiate between patients with a terminal dentition and those who are fully edentulous.

**Figure 3:**
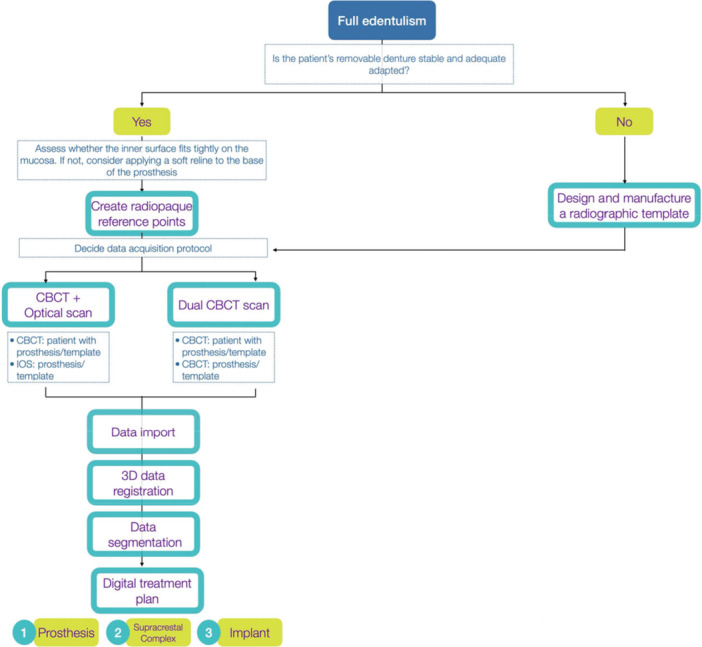
Workflows for digital implant treatment plan in full edentulism. CBCT, cone‐beam computed tomograph.

**Figure 4:**
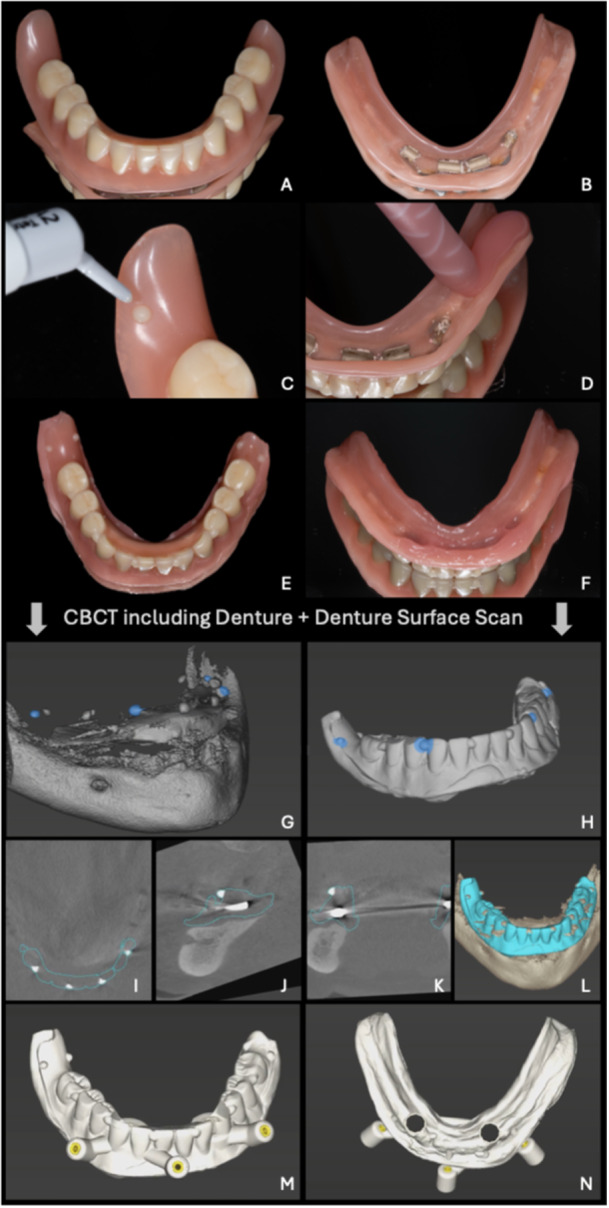
Clinical and radiographic workflow enabling digital implant planning in fully edentulous patients with adequate existing dentures. Illustrated is the case of a fully edentulous patient after implant loss from a bar‐supported hybrid prosthesis. A, B: Occlusal and basal views of the existing denture, including the metal framework corresponding to the previous bar reconstruction. C: Creation of radiopaque reference markers. D: Relining of the denture base. E, F: Occlusal and basal view of the relined denture including reference markers. G, H: Digital data registration (superimposition) and critical evaluation in axial (I), sagittal (J), coronal (K), and 3D (L) views. M, N: After virtual implant positioning, the denture surface scan was incorporated into the surgical guide design. CBCT, cone‐beam computed tomograph.

**Figure 5:**
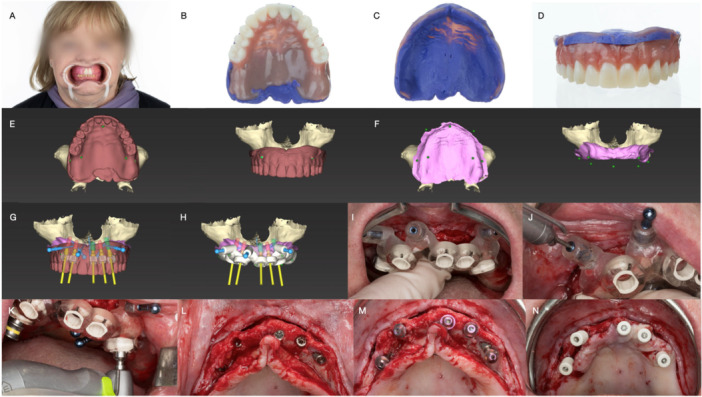
Clinical and radiographic workflow enabling digital implant planning in fully edentulous patients with inadequate existing dentures. (A) Analog mock‐up of a removable denture to determine esthetic and occlusal parameters; denture stability and adaptation of the intaglio surface should also be ensured. (B) Occlusal, (C) basal, and (D) buccal views of the new denture after relining and placement of radiographic markers. (E, F) Digital data registration (superimposition) and critical evaluation. (G) Virtual implant position planning. (H) Surgical guide design following virtual implant positioning. (I) Clinical presentation after surgical guide manufacture. (J) Anchor pin drilling. (K) Guided drilling and implant placement through the sleeves. (L) Implants in place. (M) Connection of transmucosal abutments. (N) Placement of scan bodies to determine implant position for fabrication of an immediately loaded fixed prosthesis.

**Figure 6:**
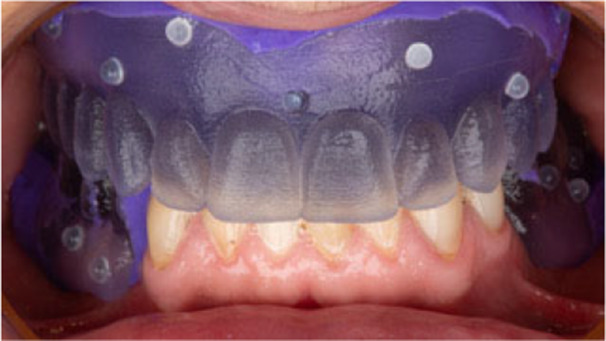
Radiographic template designed in a case of a fully edentulous patients with inadequate existing dentures.

‒ Terminal dentition
  The presence of terminal teeth might be an advantage, as they can serve as landmarks facilitating easier 3D data registration and intraoperative surgical guide support. However, in cases of extended edentulous spans, the use of registration devices, such as radiopaque set‐ups, or existing removable dentures with an adequate base and radiographic markers should be considered (Lanis et al. 2015).
‒ Edentulous patient
  The absence of teeth, and thus reliable reference structures, combined with the inherent limitations of intraoral scanning in fully edentulous patients, impairs both dataset superimposition during implant planning and the accuracy of CAIS. Consequently, the use of radiopaque registration devices during CBCT acquisition is essential to ensure a feasible and predictable workflow. The literature describes various strategies, such as bone‐supported or adhesive radiopaque markers placed directly on the edentulous ridge (Laleman et al. 2016), as well as the “dual‐scan technique” (Witherington et al. 2017). However, modern workflows have substantially simplified these procedures. Figure 3 summarized the main protocols available for digital implant treatment plan in full edentulism.
  1. In patients with adequate and stable removable dentures, an adequate number of adhesive radiopaque markers are temporarily placed on the prosthesis, which is then digitized using an optican scan (intraoral scanner or a laboratory scanner) or a CBCT scan and subsequently worn during CBCT acquisition of the patient (Figure 4).
  2. In patients with inadequate or unstable removable dentures, a radiographic template (radiopaque setup) which accurately reflects the contour of the future prosthesis should be designed, manufactured and used during CBCT acquisition. Figure 5 illustrates a case in which a new temporary removable denture was fabricated. In contrast, Figure 6 shows a radiographic template with radiopaque markers. Both approaches are valid options for digitally planning implant positioning in cases where the patient does not have an adequate removable denture.

In both scenarios, the corresponding reference points can be selected on the STL/PLY and DICOM data sets during data registration, similar to the workflow used in partially edentulous patients.

The workflow for an edentulous patient and adequate removable dentures consists of the following steps (Figure 4):

1. Verification of sufficient prosthesis design and fit. If needed, perform soft relining of the base of the prosthesis.
2. Creation of a sufficient number of radiopaque references on the existing denture, for example, using flowable composite, avoiding metal‐framework areas, and ensuring they do not interfere with the occlusion.
3. Digitalization of the denture:
  a. CBCT scan of the prosthesis, including radiopaque reference points
  b. Surface optical scan of the denture, including radiopaque reference points.
4. CBCT scan of the patient, wearing the registered denture in maximum intercuspidation during image acquisition.
5. Data import: Data are imported into the CAD‐IPS software for implant planning.
6. Data Segmentation: Performed in the same manner as in the partial edentulism workflow.
7. 3D data Registration: Accurate alignment of the DICOM and STL/PLY or DICOM files within one coordinate system. This process is typically facilitated by the CAD software by identifying at least three identical landmarks/fiducial points (i.e., radiopaque reference points) in both images.
8. Digital treatment plan: Performed in the same manner as in the partial edentulism workflow.

### 1.3 Surgical Guide Design and Execution of Implant Surgery Under NG‐ and S‐CAIS Protocols

#### 1.3.1 Non‐Guided CAIS

Non‐guided CAIS is often reported as “freehand” (Jorba‐García et al. 2023), “mental” (Chen et al. 2018), or “brain‐guided” (Afrashtehfar 2021) implant surgery, but this terminology can be misleading, and one must be aware that the use of dynamic navigation is also a “freehand” surgical procedure, albeit using optical tracking aids for navigation. Thus, it has to be clearly differentiated from ng‐CAIS.

In ng‐CAIS, the operator aims to achieve the planned implant position in the patient's arch by considering various intraoral reference points or anatomical landmarks, such as distances between adjacent teeth/implants and occlusion. This process could be further facilitated with the use of intraoperative “prosthetic templates.” These devices do not usually derive directly from the digital treatment plan, but typically offer the optimal crown dimensions and morphology as a reference for the surgeon during the osteotomy and implant placement. Thus, the term “non‐guided CAIS” was recently selected to describe this approach, as the surgeon benefits from digital tools during the planning phase, but relies on clinical experience and judgment during the execution (Jorba‐Garcia et al. 2025).

#### 1.3.2 Static CAIS

The Static Computer‐Assisted Implant Surgery (s‐CAIS) approach supplements the computer‐assisted design (CAD) with computer‐assisted manufacturing (CAM). This approach utilizes a CAM surgical guide with support and fixation elements and precisely positioned guide‐holes, which can guide specially designed drills for the preparation of the osteotomy, followed by the implant placement in the pre‐planned position (Table 1).

**Table 1:** Summarizes the different types of guides that could be produced.

Classification	Types	Definition
Support (depending on the tissue which offers support/stability)	Tooth supported	Surgical guide supported only by natural teeth.
	Bone Supported	Surgical guide supported exclusively by bone (after flap reflection). Can be with or without fixation elements.
	Mucosal Supported	Surgical guide supported exclusively by mucosa.
	Implant Supported	Surgical guide supported exclusively by existing implants.
	Hybrid supported	Surgical guide supported by different tissue or structures. Usually Kennedy class I edentulism is supported by teeth and mucosa; or mucosal supported guides in fully edentulism aided with bone anchor pins.
Design (extent of tissue coverage)	Full extension	Surgical guide covering all teeth of the arch up to the equator line.
	Partial extension	Surgical guide covering adjacent teeth of the edentulous site. It is recommended to cross the midline to give more support and stability to the surgical guide.
	With inspection windows	Surgical guide for partial edentulism, including buccal inspection windows in the guide material to allow visual verification of the intraoperative fit
Manufacturing	Milled	Surgical guide manufactured by a subtractive technique.
	3D rapid prototyping/printing:	Surgical guide made by an additive technique In‐house In‐lab
Guidance	Pilot‐drill/Partially guided	Surgical guide supporting only part of the osteotomy/implant placement protocol.
	Fully guided	Surgical guide supporting both the entire osteotomy and the implant placement.
Number of guides	Single guide	One‐piece surgical guide for the entire planned procedure
	Multiple interlocking/stackable guides	Multiple interlocking parts, corresponding to different procedures in the surgery
	Multiple non‐interlocking/stackable guides	Multiple independent guides/parts, corresponding to different procedures in the surgery
CAIS system/surgical instruments	Sleeve in sleeve/using drill handle	A drill and corresponding drill handle of varying diameters, that fit into the surgical guide's sleeve during drilling.
	Mounted sleeve‐on‐drill	A drill with a separate component that is mounted onto the drill (often clipped or screwed on) before use, hence no drill handle is needed.
	Integrated sleeve‐on‐drill	A drill where the guiding part is built into the drill as a single, fixed unit (one‐piece design aka “barrel drill”), hence no drill handle is needed
Sleeve/cylinder	No sleeve/Sleeveless	Surgical template design with guide‐hole dimensions, that correspond to the surgical instruments (i.e., without a manufacturer sleeve in the guide hole).
	With sleeve	Surgical guide designed with guide‐hole dimensions, to include a sleeve by the manufacturer.
	Open sleeve	Surgical guide designed with guide‐hole dimensions, to include a sleeve by the manufacturer. This sleeve has a fenestration in the buccal aspect to allow for improved irrigation and access in patients with limited mouth opening.
Sleeve material	PEEK Sleeve	
	Metal sleeve	
	Zirconia Sleeve	

Abbreviation: CAIS, computer‐assisted implant surgery.

#### 1.3.3 Workflow in Partial Edentulism

**Figure 7:**
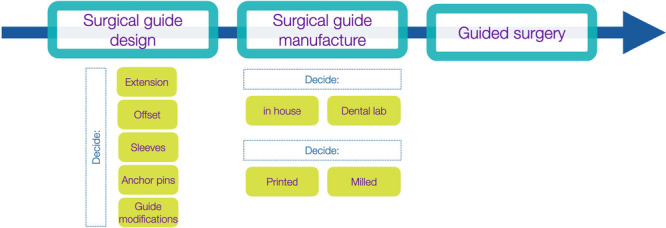
Static computer‐assisted implant surgery (CAIS) workflow.

Static CAIS workflow is summarized in Figure 7 and depicted in a clinical case in Figure 2:

1. Surgical guide design: Usually, within the same CAD‐IPS, a device including guide holes corresponding to components of the CAIS system is designed based on the patient's anatomic local features. Several parameters must be defined, such as the extent of the surgical guide, the use of sleeves, the offset to supporting structures, the use of additional bone support (i.e., anchor pins), or the inclusion of buccal fenestrations to verify the correct fit of the surgical guide during the surgery. Once the guide design is validated, the file is exported as an STL file.
2. Surgical guide manufacture: The actual guide can be manufactured using an additive (3D printed) or subtractive (milled) CAM procedure. 3D Printing can usually be performed in‐house (in the dental office) or in the dental lab. Once the surgical guide is manufactured, specific metallic sleeves can be inserted into the access holes.
3. Guided Surgery: After guide insertion and fit verification, the implant osteotomy and placement are conducted according to the respective Guided Implant Surgery Protocol (GISP). This involves the use of special implant drills and other components included in the Guided Implant Surgery Kit. During GISP, the clinician should not blindly trust the procedure and must meticulously validate the correct transfer of the virtual treatment plan to the real world, for example, by using depth gauges, conventional intraoperative measurements, and if deemed essential also taking radiographs.

#### 1.3.4 Workflow for Immediate Implant Placement

For implant placement in post‐extraction socket, the “virtual tooth extraction” is usually conducted automatically at the CAD‐IPS when a surgical guide is designed and the tooth to be extracted is designated. Thus the mesh of the optical scan is modified to remove the crown of the respective tooth. In case the CAD‐IPS does not allow to automatically modify the mesh in the implant site, third party software must be used to eliminate the tooth/teeth from the patient's optical scan.

#### 1.3.5 Workflow in Full Edentulism

Pre‐edentulous/terminal dentition patient.

As mentioned in Phase B, “Implant Treatment Planning,” in patients with hopeless dentition, the presence of remaining teeth can assist clinicians during the planning and surgical phases. This situation is treated in a workflow combining immediate implant and edentulous workflows.

1. Modification of the mesh: This step follows the workflow of immediate implant placement. Special attention must be given during the modification of the STL/PLY mesh, as certain teeth—if not located in the implant site—may be intentionally preserved in the initial file and not digitally extracted initially, to provide support for the surgical guide. Therefore, at this stage, clinicians must decide whether to extract all planned teeth at the beginning of the procedure prior to implant placement, thereby designing a mucosa‐ or bone‐supported guide, or to perform only selected extractions beforehand, allowing the use of a hybrid‐supported guide and leaving certain extractions for after implant placement.
2. Perform implant planning as described in the Computer‐Assisted Design—Implant Therapy Plan section for full edentulism.
3. Surgical guide design: Design the surgical guide as in the partially edentulism workflow, using the modified STL/PLY file without the teeth to be extracted in the future implant sites. Special attention should be paid to the design, number and position anchorage elements (i.e., anchor pins) if needed. Once the guide design is validated, the design is exported to an STL file.
4. Surgical guide manufacture: To be performed in the same manner as in the partial edentulism workflow.
5. Surgical phase: To be performed in the same manner as in the partial edentulism workflow.

Edentulous patient

Clinical cases and workflows are illustrated in Figures 4 and 5:

1. Perform implant planning as described in the Computer‐Assisted Design—Implant Therapy Plan section for fully edentulism.
2. Surgical guide design: Design the surgical guide as in the partially edentulism workflow.
3. Surgical guide manufacture: To be performed in the same manner as in the partial edentulism workflow
4. Surgical phase: To be performed in the same manner as in the partial edentulism workflow.

### 1.4 Time‐ and Cost‐Effectiveness

The benefits and limitations of the use of CAIS with respect to accuracy (Mahardawi et al. 2025) have been extensively investigated (Siqueira et al. 2020; Jorba‐García et al. 2021; Takács et al. 2023; Tahmaseb et al. 2018; Bover‐Ramos et al. 2018). However, clinical outcomes (Sadilina et al. 2025), patient‐reported outcomes (Yeo et al. 2025b), clinician‐reported outcomes, as well as the implications for education and training (Uei et al. 2025), remain comparatively underexplored. The cost‐effectiveness of CAIS has also received limited attention, despite the fact that financial considerations represent a major barrier for the adoption of these workflows in daily clinical practice and may even influence patient acceptance (Lukkanasomboon et al. 2025).

**Figure 8:**
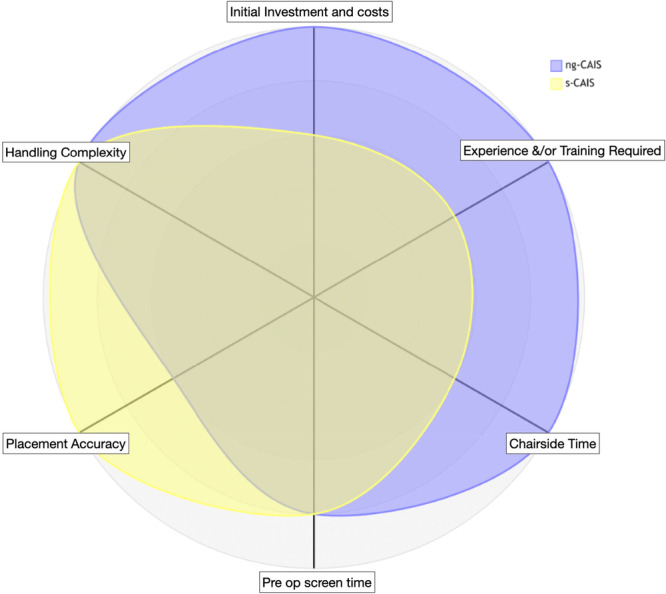
Radar chart of the main cost/effectiveness drivers when comparing ng‐ (blue) and s‐CAIS (yellow) in the case of replacement of one tooth with a single implant‐supported prosthesis. Each factor is scored on a scale from 1 (lowest) to 5 (highest). This visualization illustrates that ng‐CAIS (5/5) requires extensive clinical experience, while s‐CAIS (3/5) relies on training on the guided surgery protocol. Both approaches can handle the complexity of a straightforward single implant effectively when performed by a trained clinician. Regarding chairside time, ng‐CAIS (5/5) is fastest, as it requires no guide placement or guided surgery kit setup, and also has only baseline costs. This visualization supports that for straightforward cases, ng‐CAIS remains a valid, cost‐effective option, while s‐CAIS provides superior accuracy for clinicians who prioritize accuracy over efficiency. CAIS, computer‐assisted implant surgery.

**Figure 9:**
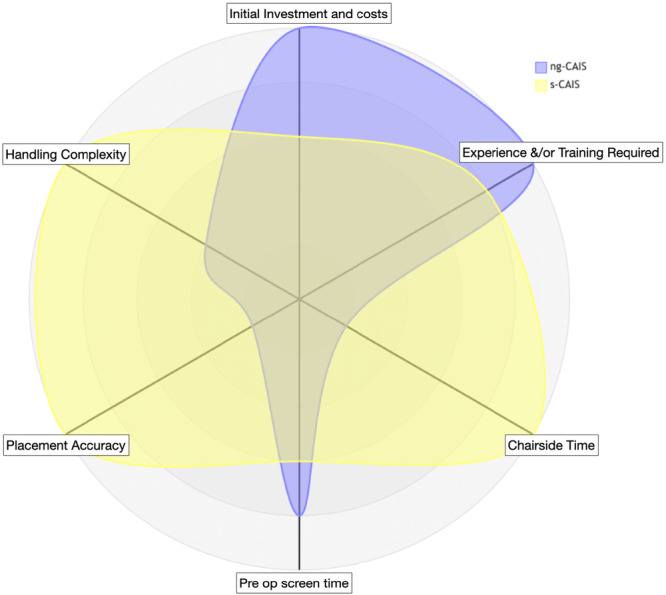
Radar chart of the main cost/effectiveness drivers when comparing ng‐ (blue) and s‐CAIS (yellow) in the case of edentulous patients. Each factor is scored on a scale from 1(lowest) to 5 (highest). This visualization illustrates that ng‐CAIS (5/5) requires extensive clinical experience, but the application of s‐CAIS (4/5) in this case also demands training in a guided surgery protocol of higher complexity. At the same time, a guided surgery s‐CAIS protocol can facilitate the complexity of the edentulous arch much more effectively than ng‐CAIS when performed by a trained clinician. Likewise, s‐CAIS is anticipated to result in faster implant surgery, despite the additional time in placement/fixation of the surgical guide. Investment and costs remain lower with ng‐CAIS. This chart illustrates the significant trade‐offs in edentulous cases, where ng‐CAIS only excels in cost reduction and requires somewhat less pre‐op planning time, but lacks significantly in accuracy, chairside efficiency, and handling complexity. The visualization suggests s‐CAIS is essentially mandatory for predictable full‐arch implant rehabilitation, despite its higher upfront costs and planning requirements. CAIS, computer‐assisted implant surgery.

Overall, CAIS is associated with increased costs during both the treatment‐planning and surgical execution. Nevertheless, by promoting prosthetically driven implant positioning, CAIS has the potential to simplify subsequent prosthodontic workflows and, in some cases, reduce prosthesis‐related costs and chair time, particularly when optimal implant placement enables the use of stock components instead of custom‐made prosthetic parts. Likewise, whether CAIS modalities contribute to more efficient use of clinical time, and under which circumstances, remains a matter of ongoing debate. Assessing the main cost drivers in ng‐ and s‐CAIS it is reasonable to assume that ng‐CAIS remains a valid, cost‐effective option for single gap and straightforward cases (Figure 8), while s‐CAIS is essentially mandatory for predictable full‐arch implant rehabilitation (Figure 9), despite its higher upfront costs and planning requirements.

#### 1.4.1 The Digital Treatment Plan

The main cost drivers associated with implementing the digital treatment plan required for CAIS can be summarized as follows:

A. Hardware:
  If not acquired from an external radiology center, an initial investment is required to acquire a CBCT unit and an intraoral optical surface scanner. Although a CBCT system represents a substantial financial commitment, its diagnostic value across multiple clinical domains beyond implantology may justify its integration into modern dental practice. In addition, the increasing availability and competitive pricing of IOS have made these devices progressively more accessible.
B. Software
  The acquisition of a CAD‐IPS constitutes another major cost component. Various software solutions are currently available, each offering different functions, limitations, and licensing models, which makes the cost‐effectiveness evaluations complex. Most platforms require recurring fees for license purchase or renewal, while others permit free planning but charge a fee for exporting the digital treatment plan for the manufacturing of surgical guides or prosthetic components. This latter “pay‐as‐you‐go” model may be particularly advantageous for clinicians beginning their adoption of CAIS or for practices with a low volume of implant cases.

Interoperability between CAD‐IPS solutions and other essential software in the digital workflow represents an important consideration and often complicates decision‐making. Because no single solution is universally optimal at present, clinicians are encouraged to thoroughly evaluate available options and select software that aligns with the complexity of their implant therapy needs and the business model best suited to the volume of implant patients treated in their practice.

As a general consideration, the following aspects should be evaluated before deciding which CAS‐IPS software is most suitable for a clinician's expectations:

- User‐friendly and intuitive interface, especially for beginners.
- Availability of different implant brands and Guided Implant Surgery Systems (GISS).
- Software licensing and pricing: Several modalities exist:
  ◦ Annual licenses that allow full functionality and case exports.
  ◦ Free software with a fee per export (often with packages for multiple exports).
  ◦ Modular software that allows free visualization and implant planning but requires purchasing a software extension to design the surgical guide.
- Extent of application of AI within the software, (e.g., data segmentation, data registration, and planning).
- Ability to design virtual mock‐ups and prostheses.
- Ability to add multiple STL layers, particularly useful in complex cases.
- Flexibility in surgical guide design, allowing modification of aspects such as sleeve offset, guide extension, guide openings to ensure correct positioning, and the possibility of designing advanced guides such as stackable guides.

#### 1.4.2 Non‐Guided CAIS

The cost associated with ng‐CAIS is the lowest among all four CAIS approaches. While expenses related to the digital treatment plan remain, the absence of guide or component fabrication allows clinics to rely on open‐access CAD‐IPS platforms offering free planning services. In this scenario, the production of intraoperative templates requires an analog setup, incurring only the corresponding laboratory costs.

However, evaluating the cost‐effectiveness of ng‐CAIS remains challenging. The comparatively lower accuracy of implant placement may substantially prolong, or even preclude, complex procedures such as flapless surgery or immediate loading, particularly in edentulous patients. Hence, determining the true cost implications of ng‐CAIS requires a broader assessment that encompasses not only isolated surgical performance but also workflow‐level clinical outcomes and patient‐reported and clinician‐reported experiences (Yeo et al. 2025b).

#### 1.4.3 Static‐CAIS

a. Hardware
  Acquisition and maintenance of a Guided Implant Surgical Kit (GISK) entail both initial and ongoing costs, although these are generally modest. As these kits are typically system‐specific, the use of multiple implant systems necessitates maintaining separate GISKs and associated components, increasing inventory complexity and imposing additional training requirements for auxiliary staff. Following digital treatment planning, clinicians may choose either in‐house manufacturing of the surgical guide or outsourcing this process to a dental laboratory.
  Surgical guides are most commonly produced via 3D printing, whereas milled guides are generally more expensive.
  When outsourcing to a dental laboratory, transport and logistical costs must also be considered. For in‐house guide fabrication, a substantial initial investment his required. This includes the acquisition of a dental‐grade 3D printer (e.g., Nexdent, SprintRay, or Formlabs) or a generic alternative (e.g., Anycubic or Phrozen). Costs vary depending on the brand, printing accuracy, and build platform size. Additional expenses include printing resins. Particular care is needed, especially with generic 3D printers, to ensure the use of biocompatible resins approved for intraoral applications. Moreover, printer calibration must be performed according to the specific resin used. Dental‐specific printers generally offer more streamlined and user‐friendly calibration workflows.
  Once the initial investment is made, in‐house production incurs only the ongoing costs of electricity, printer maintenance, staff time, and consumables, such as resins. Manufacturer‐provided metal or plastic sleeves, typically purchased separately, contribute to the per‐guide cost.
b. Software
  Beyond the CAD‐IPS used for digital treatment planning, no additional software is strictly required for s‐CAIS. However, licensing fees are often applied for exporting the guide design for 3D printing. For in‐house manufacturing, software associated with operating the 3D printer is also necessary.

#### 1.4.4 Time‐Effectiveness

The time‐effectiveness of ng‐ and s‐CAIS is difficult to evaluate and is likely best assessed within specific clinical scenarios. The clinicians' time commitment is influenced not only by the protocols, devices and workflow used, but also by the clinician's familiarity and expertise with the system (including time for training and education), the volume and complexity of implant cases and the organizational setup of the clinic, laboratory and dental team.

In general, the time required for data acquisition, including the intraoral scanning, tends to be more efficient than that of corresponding analog workflows, and digital data management, transfer, storage and retrieval offers substantial advantages. However, digital treatment planning demands a considerable time investment, both in terms of initial training and on a case‐by‐case basis, especially when treating complex patients. This burden may increase further when multiple software platforms are required due to limitations in functionality or interoperability. Within a digital workflow, “screen time becomes patient time,” a shift that may not be welcomed by all clinicians. Many still prefer to devote their time to chairside procedures rather than computer‐based tasks, which has contributed to the growing use of third‐party digital planning services offered by implant manufacturers, dental laboratories, or specialized companies.

While these services can streamline workflow, they also increase treatment costs and introduce professional, ethical, and practical considerations related to ownership of the digital treatment plan and the distribution of responsibility and liability among involved parties. The integration of artificial intelligence–assisted functions into CAD‐IPS platforms further blurs these boundaries. Consequently, the domain of digital treatment planning remains a rapidly evolving landscape, with many key parameters yet to be fully defined.

## 2 Discussion

Implant Dentistry is currently experiencing a significant paradigm shift from conventional to digital workflows, in which a fully virtual, comprehensive treatment plan, visualized and planned in its entirety, is replacing the traditional sequential approach to surgical and restorative procedures. This transition brings along many implications: while it can simplify the execution of surgical procedures, it also demands greater effort and attention during the digital treatment planning phase. Concurrently, a new workflow is emerging that integrates diverse software, hardware, and procedural steps, extending beyond the chairside time with the patient. Although these workflows are now well established, their implementations are not always seamless; integrating essential software and hardware tools often presents substantial challenges for clinicians. A thorough understanding of the sources of complexity in each workflow is therefore essential to identify bottlenecks and improve efficiency through simple and intuitive protocols.

The digital treatment plan is increasingly recognized as the fundamental starting point of any CAIS workflow. Rather than serving as a loose guide toward clinical outcomes, it functions as a comprehensive visualization of an entire system, capable of orchestrating complex treatment protocols with precision, including immediate or flapless surgical approaches. While this represents a powerful clinical tool, it also imposes a dual responsibility on the clinician: first, to master the essential biological principles underlying sound, patient‐centered implant planning; and second, to effectively leverage digital tools to implement these principles in daily clinical practice.

In support of the first responsibility, a highly active research field has emerged, aiming to identify how design elements and prosthetics influence the peri‐implant mucosa (Rungtanakiat et al. 2024) and the surrounding peri‐implant bone (Strauss et al. 2024). This body of work complements the well‐established knowledge regarding surgical placement and implant positioning in relation to the local anatomy, distance between adjacent implants or teeth, the depth of the implant platform, its angulation, and the bucco‐lingual positioning of the implant (Buser et al. 2004; Testori et al. 2018; Jivraj and Chee 2006; Zitzmann et al. 1999). It is evident that the understanding of the biological and restorative principles underlying sound implant design has advanced considerably in recent years (Pedrinaci et al. 2024a). Nevertheless, this progress has simultaneously increased the clinician's responsibility to apply these principles effectively.

At the same time, leveraging digital tools to develop a virtual implant treatment plant presents its own set of challenges. Despite the extensive use of 3D imaging, determining the ideal implant position on a two‐dimensional (2D) computer screen remains difficult and demands training and experience. To enhance digital implant planning on a computer screen, the use of multiple non‐orthogonal CBCT planes can aid in determining a 3D patient‐optimized implant position. This approach, combined with 3D CBCT reconstructions, surface scans, and a 3D virtual mock‐up, could allow for more accurate and comprehensive treatment planning even when working on a 2D display.

Accurate interpretation of complex anatomical and prosthetic relationships on a flat interface relies on well‐developed spatial reasoning skills (Yao et al. 2019), an essential, yet often overlooked, competency. Deficiencies in this skill can lead to variability in implant positioning, even when advanced software tools are employed.

A recent study highlighted this issue by evaluating intra‐operator consistency in virtual single‐implant planning. When the same clinicians planned the same case twice, with a washout period in between, deviations between the two plans reached 3.28 ± 1.99° in angulation, 0.78 ± 0.46 mm at the implant platform, and 1.12 ± 0.61 mm at the apex, despite being performed by the same individual. Furthermore, when comparing these plans to a predefined gold standard, inter‐operator deviations averaged 3.8 ± 1.94° angularly, 1.11 ± 0.55 mm at the crestal level, and 1.54 ± 0.66 mm at the apical level. These findings demonstrate both intra‐ and inter‐operator variability in implant planning, underscoring the inherent challenges of defining an optimal implant position, even when comprehensive 3D anatomical data, such as CBCT and STL files, are available (Raabe et al. 2024).

This variability raises an important and thought‐provoking question: could clinicians, in some cases, achieve better implant positioning when using a non‐guided approach compared to their own digitally planned positions? Such considerations emphasize the complexity of treatment planning in digital implantology and highlight the need for continued evaluation of the accuracy and clinical relevance of virtual plans. At the same time, it is important to recognize that the “optimal” implant position is rarely a single precise point; rather, it can often be conceptualized as a range or a zone, commonly referred to as a “comfort zone,” that accommodates slight variations in positioning.

Interestingly, when evaluating the influence of prosthetic‐driven planning for single implants, the inclusion of a virtual crown or diagnostic mock‐up altered the planned implant position in approximately 50% of cases (Raabe et al. 2024). This finding is not straightforward to interpret. Despite growing evidence that restorative design can affect long‐term peri‐implant tissue stability (Janda and Mattheos 2024), there remains limited consensus on essential design. Clinicians regardless of experience level, may differ in their understanding of prosthetically‐driven treatment planning and may adopt different approaches. In some cases, surgical considerations may take precedence due to anatomical limitations. Additionally, in straightforward single‐tooth cases, the added value of a virtual mock‐up may have less influence on implant positioning. Nevertheless, it is clear that even when prosthetic information is incorporated, variability in implant planning remains a significant consideration. Given the critical importance of the digital treatment plan, translating current evidence into practical, easily implementable guidelines is essential.

Emerging technologies have the potential to substantially improve both the process and outcomes of the digital treatment plan. Recent research has explored the use of mixed reality (MR) technology for implant planning through holographic visualization. In this innovative approach, clinicians can interact with and manipulate 3D models using an MR headset that projects holographic representations of the patient's anatomy. This enables treatment planning in a true 3D environment, rather than being confined to a conventional 2D screen (Mangano et al. 2024). Furthermore, the integration of artificial intelligence (AI) promises to streamline and enhance the Computer‐Assisted Design (CAD) phase of implant planning. AI algorithms can perform 3D data registration with high precision, often surpassing the accuracy of manual, human‐based alignment. In addition, AI‐driven segmentation of anatomical structures appears to be both efficient and reliable, at least when it comes to bone, natural teeth, and surrounding anatomic structures (Fernanda et al. 2025). The incorporation of AI in prosthesis design and the overall treatment planning could reduce the clinician's burden of repetitive tasks and screen time. However, these technologies are not without risks and limitations. Machine Learning algorithms trained on large datasets of previous cases may inadvertently perpetuate outdated or suboptimal designs, rather than implementing the latest evidence‐based principles. Therefore, all such technologies and algorithms must undergo extensive evaluation across diverse clinical scenarios before widespread implementation in routine daily practice (Elgarba et al. 2024b; DeNucci et al. 2025).

This article covers a broad spectrum of digital implantology concepts, some of them of high complexity. This may at times limit the depth of discussion and detail provided. While the paper highlights key concepts, technologies, and considerations, it serves as a starting point in clinicians' digital journey and does not replace dedicated resources and or advanced training for clinical implementation.

In conclusion, non‐guided CAIS approaches remain relevant and cost‐effective for straightforward treatment protocols, where a higher tolerance for deviation is acceptable. Static‐CAIS, is currently the most widely used workflow for guided implant placement, offering extensive documentation of predictable outcomes and high implant positioning accuracy.

Despite the wide adoption of s‐CAIS protocols, friction points persist within the workflow, and the inherent limitations of surgical guides and the guided implant surgery kits may reduce their utility.

Although emerging technologies such as dynamic and robotic CAIS are increasingly applied in implant surgery, s‐CAIS is expected to remain the dominant guided approach for guided implant surgery in the near future (Yeo et al. 2025a). Therefore, streamlining and improving s‐CAIS workflows, while enhancing education and training standards, could have a significant impact on achieving better treatment outcomes on a global scale.

## Author Contributions

**Adrià Jorba‐Garcia:** conceptualization, investigation, writing – original draft. **Emilio Couso‐Queiruga:** investigation, writing – review and editing. **Clemens Raabe:** investigation, writing – review and editing. **Krzysztof Chmielewski:** investigation, writing – review and editing. **Michael Bornstein:** investigation, writing – review and editing. **Bilal Al‐Nawas:** supervision, writing – review and editing. **Nikos Mattheos:** conceptualization, writing – original draft, writing – review and editing supervision.

## Funding

The authors received no specific funding for this work.

## Conflicts of Interest

The authors declare no conflicts of interest. Adrià Jorba‐Garcia and Bilal Al‐Nawas, have received travel grants from the International Team for Implantology (ITI) for attendance of the meetings related to the writing of the paper.

## Data Availability

Data sharing is not applicable to this article as no new data were created or analyzed in this study.
